# Review of Indications for Endotracheal Intubation in Burn Patients with Suspected Inhalational Injury

**DOI:** 10.3390/ebj4020014

**Published:** 2023-03-29

**Authors:** Elizabeth Concannon, Lindsay Damkat Thomas, Lachlan Kerr, Ivo Damkat, Benjamin Reddi, John E. Greenwood, Nicholas S. Solanki, Marcus J. D. Wagstaff

**Affiliations:** 1 Adult Burn Service, Royal Adelaide Hospital, Adelaide, SA 5000, Australia; 2 National Burn Service, Middlemore Hospital, Auckland 2025, New Zealand; 3 Independent Researcher, Auckland 1072, New Zealand; 4 Department of Critical Care and Anaesthesia, Royal Adelaide Hospital, Adelaide, SA 5000, Australia

**Keywords:** inhalation injury, intubation criteria, intubation, bronchoscopy, burn injury, ABA criteria, Denver criteria, early extubation

## Abstract

Inhalation injury is a major contributor to mortality following burn injury. Despite recognised clinical criteria to guide the intubation of burn patients, concerns remain regarding overutilisation of intubation. Complications can arise as a result of intubation, including ventilator-associated pneumonia (VAP). This study reviews the indications for intubation against the internationally accepted criteria (American Burns Association (ABA) and Denver criteria) for burn patients treated at the Royal Adelaide Hospital (RAH) burns unit between 2017 and 2020. Burn patients who were intubated on arrival to the RAH or in a pre-hospital setting were identified using the BRANZ database. Indications for intubation were compared to the ABA and Denver criteria. A total of 61 patients were identified with a mean total body surface area of 17.8%. A total of 95% of patients met one of the ABA and Denver criteria. The most common ABA and Denver criteria for intubation was deep facial burns or singed facial hair, respectively. Most adult patients with burns admitted to the RAH are intubated per published criteria. Early nasoendoscopy/bronchoscopy may be useful in determining patients who can be safely extubated within 48 h.

## 1. Introduction

Burn injury accounts for an estimated 180,000 deaths per annum worldwide [1]. In Australia, burns account for 1% of hospitalised injury cases, and carries a mortality of 0.8% annually [2]. Inhalation injury represents one of the strongest predictors of mortality in burns [3]. Despite advances in critical care, the mortality rate in burn patients with inhalation injury is reported to be 10–30%, a rate that increases with increasing total body surface area burned (TBSA) and increasing age.

Inhalational injury can be defined as the toxic and deleterious effects from heat and chemical products of combustion on the airway lungs and systemic health [4]. The prevalence of inhalation injury in burn patients varies in published literature from 5% to 35% [5]. Recent Australian research [6] demonstrated that 40% of patients who had a burn >20% TBSA had evidence of inhalation injury.

An aggressive early approach to endotracheal intubation of patients with suspected inhalation injury can be justified by the potentially catastrophic consequences of loss of airway control due to upper-airway oedema that may develop over the first 48 h post-burn. Intubation is not a benign procedure and places patients at risk of trauma to the oropharyngeal cavity, trachea, or vocal cords; aspiration and even death due to unrecognised oesophageal intubation [7]. Prolonged mechanical ventilation can predispose the patient to tracheal stenosis, dysphagia, dysphonia, ventilator-associated pneumonia, ARDS, failure to wean, delirium, and critical illness polyneuropathy [8]. Subsequent health economic impacts of prolonged mechanical ventilation include increased length of intensive care stay and burden associated with prolonged rehabilitation [9,10]. Timely extubation is equally as important in burn management protocols, allowing patients to be transferred from intensive care units back to the burns unit for specialised multidisciplinary care and early rehabilitation.

Two scoring tools available to guide appropriate early intubation of burn patients are the American Burns Association (ABA) intubation criteria [9] and the Denver Criteria [10] (Table 1). The latter includes two additional criteria to increase the sensitivity of the former for inhalational injury. Despite intubation scoring tools, a proportion of patients who would never have developed a threatened airway may be intubated and concerns remain regarding over-intubation [11]. Teaching courses and educational material, such as the Emergency Management of Severe Burns, must also be considered as a cautious approach aiming to avoid emergency intubation.

There is a lack of consensus regarding diagnosis, grading of severity or prognosis of inhalation injury [12]. Direct laryngoscopy and flexible nasoendoscopy are useful tools for upper-airway examinations. Bronchoscopy assessment can be used as a tool to diagnose lower-airway inhalational injury [4,13]. Additionally, bronchoscopy findings assist with the selection of patients who can be safely managed without intubation or determine the appropriateness of early extubation [14]. Bronchoscopy is not a universally available modality, particularly in pre-hospital and pre-burn centre settings. Clinical scoring tools, though non-specific, have a more practical role in the initial acute assessment of suspected inhalational injury.

### Aims

The primary objective of this study was to review indications for endotracheal intubation and measure concordance with the ABA and Denver clinical criteria in burn patients treated at the study site (Royal Adelaide Hospital) over a 3-year study period, from 2017 to 2020. A secondary aim was to correlate indications for intubation with evaluation of bronchoscopic findings, where performed.

## 2. Materials and Methods

All adult burn patients admitted to the Royal Adelaide Hospital (RAH) between 2017 and 2020 who underwent endotracheal intubation on or prior to arrival, for suspected inhalation injury, were identified using the Burns Registry of Australia and New Zealand (BRANZ). BRANZ is a prospectively maintained clinical registry capturing epidemiological, quality of care, and outcome data for burn patients across Australian and New Zealand burn units. Patients were excluded if initially intubated for an injury other than burn related injury or if they underwent active management of moderate—severe inhalational injury with Heparinised N-Acetylcysteine (HepNAC) to avoid potential bias to the analysis of our results given its beneficial effect on duration of intubation and outcomes of mechanical ventilation.

Further patient data including demographics, burn characteristics and details relating to location and indication for intubation were elicited by interrogation of an existing intensive care unit database maintained at the RAH as well as paper chart and electronic medical record review. Data were collated in an excel database to allow for descriptive interrogation using a standard statistical software package R.

Bronchoscopic findings, where performed, were reviewed and classified as per the Abbreviated Injury Score (AIS) grading scale [13,14] (Table S1) which is a 5-point grading scale of inhalation injury based on the description of findings at bronchoscopy. Fine nasal endocsocpy (FNE) was carried out on first presentation to the study centre (generally within 12 h of injury) for patients with suspected inhalational injury. Bronchoscopy was carried out at the initial surgical debridement within the first 24 h of injury and a second bronchoscopy at 24–48 h post-injury if there was ongoing concern for inhalational injury. Early extubation was defined as that lasting less than 48 h. Prolonged mechanical ventilation was defined as continuous intubation for 7 days or longer from first presentation.

Rapid shallow breathing index (RSBI) scores were calculated using patient data at time of extubation—this is a validated tool based on ratio of tidal volume (TV) to respiratory rate (RR), designed to predict successful extubation (when TV/RR score is less than 105) [15,16].

Acute Physiology and Chronic Health Disease Classification System (APACHE) II and III mortality prediction scores were calculated on the first day of intensive care unit (ICU) admission [17,18,19,20]. APACHE scores reflect a patient’s physiological response to an initial insult and the effects of resuscitation during the initial 24 h and have been shown to correlate well with mortality amongst patients with burn and inhalational injury.

Ventilator-associated pneumonia (VAP) was defined as pneumonia occurring more than 48 h after patients have been intubated and received mechanical ventilation. VAP diagnosis was by way of clinical suspicion combined with bedside examination by a critical care consultant, radiographic examination, and microbiologic analysis of respiratory secretions.

## 3. Results

A total of 64 burn patients who were intubated prior, or on arrival, to the RAH were identified through BRANZ during the 3-year study period. Three patients were excluded as they were intubated for reasons other than their burn such as concomitant head injury. Three patients were excluded due to active management of moderate—severe inhalational injury with HepNAC as per a change in management policy that came into effect during the latter 6 months of the three year study period. A further 3 patients were removed as they met neither EBA or Denver criteria leaving a final study population of 55 patients with a male preponderance. The three patients that did not meet any criteria for intubation were intubated in the operating room pre-operatively at the time of early burn debridement and did not undergo immediate post-operative extubation for a variety of reasons including the expectation for facial oedema and high analgesic requirements post-operatively.

Patient demographics are described in Table 2. Thirty-two patients (58%) were intubated prior to arriving to the RAH, with twenty-one (38%) patients being intubated in the pre-hospital setting, generally guided by phone consultation with the RAH burns service. Five patients (9%) died during the course of their hospital admission following palliation due to extensive concomitant non-burn injuries. The median length of stay (LOS) was 40 days, with 4 (91 h) of those spent in ICU, and a median of 5 days spent mechanically ventilated.

### 3.1. Concordance with ABA and Denver Indications for Intubation

**Figure 1:**
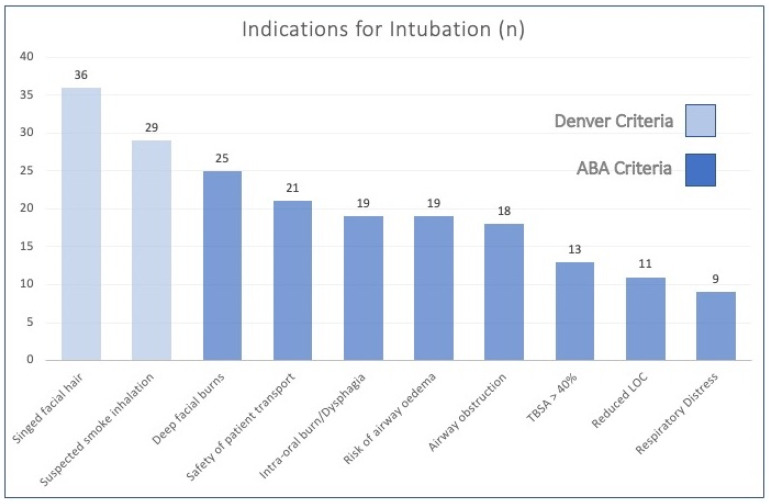
Incidence of ABA and Denver indications for intubation. LOC: level of consciousness.

A total of 76% and 100% of patients met one or more of either the Denver/ABA criteria, respectively (Figure 1 and Table 3). The mean number of criteria met for ABA listed indications was 2.2, and 1.2 for the Denver criteria.

The most common indications for intubation were represented by the two Denver criteria (Figure 1)—namely singed facial hair in 65% (*n* = 36) of patients and suspected smoke inhalation present in 53% (*n* = 29). The most common ABA criteria was deep or full thickness facial burns, identified in 45% (*n* = 25) of cases as the reason for intubation.

Risk of airway oedema and patient transport were documented as contributing factors in the decision to intubate in 19 and 21 patients, respectively, together amounting to 73% of the overall cohort. Given their prevalence, these factors may be worth including in future criteria for intubation that could be applicable for burn centres serving vast geographical catchment areas as is the case in many of the Australian New Zealand burn services.

### 3.2. Bronchoscopic Findings

**Figure 2:**
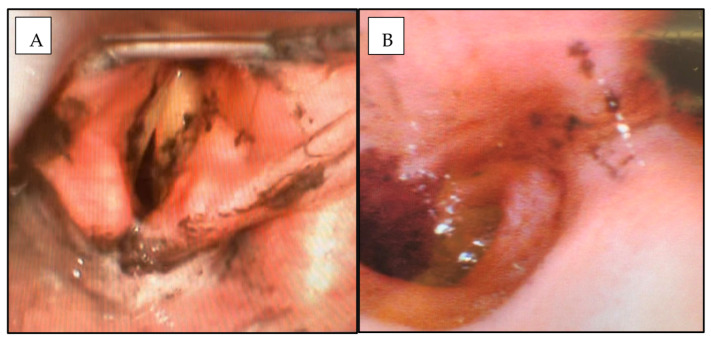
Flexible nasoendoscopy (**A**) and fibreoptic bronchoscopy (**B**) findings at Day 0 post-burn demonstrating moderate inhalational injury with erythema, carbonaceous deposits and bronchorrhea.

Twenty-four of the intubated patients (44%) underwent bronchoscopy (Figure 2 and Table 4). Bronchoscopy findings are outlined in Table 4. Despite the high proportion of patients meeting multiple ABA/Denver criteria for intubation, 16 patients, or 67%, of those who underwent bronchoscopy, had normal findings with no evidence of lower-airway inhalation injury. A total of 78 patients, 33% of those who underwent bronchoscopy, had evidence of lower-airway inhalational injury.

Seven patients (12% of the overall cohort) underwent flexible nasoendoscopy (FNE) alone (Figure 2), without proceeding to bronchoscopy. Patients with normal findings on FNE or direct laryngoscopy findings and low clinical suspicion for lower-airway inhalational injury do not generally proceed to bronchoscopy at our centre unless there is an alternative indication for this investigation. Just over half of the 55 intubated patients (31 patients or 56%) were extubated within 48 h.

Three patients were excluded from analysis as they had active management of severe (AIS Grade 2–3) lower-airway inhalational injury with nebulised N-Acetylcysteine 600 mg and Heparin 10,000 units (HepNAC), 6 hourly. This therapy is a modality that was introduced into our practice during the latter 6 months of the 3-year study period, as an adjunct to standard respiratory supportive measures for selected patients with inhalational injury. Combined nebulised fibrinolytic (Heparin) and mucolytic (N-Acetylcysteine) therapies have been reported to ameliorate acute lung injury and shorten duration of mechanical ventilation by reducing the fibrinocellular obstructive cast formation in the alveolar spaces that is considered a hallmark of smoke inhalation [21,22]. Nebulised HepNAC is now considered on a case-by-case basis at our unit, in consultation with our critical care colleagues, for all patients with bronchoscopy confirmed lower-airway inhalational injury. AIS grading can help characterize severity of inhalational injury and can help to identify patients who warrant active management with HepNAC.

### 3.3. Policy of Early Extubation and Prediction of Extubation Failure

A policy of early burn debridement (within 24 h of injury) and early extubation (within 48 h if clinically feasible) exists in the Royal Adelaide Hospital, for reasons outlined in the discussion section. As such, 56% of patients (31) were extubated within 48 h of intubation. A total of 7 patients (12%) had a failed extubation attempt, defined as inability to sustain spontaneous breathing after removal of the endotracheal tube and need for reintubation within 72 h. Causes of extubation failure and other respiratory parameters are outlined in Table 5.

The rapid shallow breathing index (RSBI), calculated as the ratio of tidal volume (TV) in litres to respiratory rate (RR) in breaths/minute, has been reported to predict ventilator weaning success [15]. A total of 7 7 patients (12%) failed extubation despite all having a mean RSBI score of 40 (range 12–63), with RSBI score < 105 reported to be associated with weaning success. The mean RSBI score was 31 (range 12–61) in the remaining 46 patients (excluding 5 patient mortalities) who underwent successful endotracheal tube extubation at a mean of 3.5 days (range 3 h to 21 days). Of the six patients in this cohort who failed extubation, two were female (Patients 1 and 7 in Table 5.) and the remainder were male. Three patients who failed extubation had ongoing suctioning measures in place for respiratory secretions due to VAP which were documented as factors that contributed to their extubation failure. Two patients (Patients 5 and 7) required prolonged ventilatory support due to neurological dysfunction with no evidence of lower inhalation injury on bronchoscopy.

## 4. Discussion

A move toward aggressive early intubation of patients with suspicion of inhalation injury has been advocated as part of the Advanced Trauma Life Support algorithm since the 1970s. This low threshold policy aims to circumvent potentially catastrophic consequences of missed inhalation injury or asphyxia from airway oedema that can be further exacerbated by large-volume fluid resuscitation used to treat burn shock. A marked increase in rate of intubations in burn patients was seen after introduction of ATLS in the Netherlands from 38% to 76% between the 1980s and 1990s [23] with no difference in incidence or degree of burn injuries during that time. Early intubation can also serve as a ‘prophylactic’ measure to minimise risks associated with out-of-hospital intubation attempts that can become necessary due to evolving airway oedema or high opiate requirements during patient transfer to major burns centres. A major challenge exists in retrospectively determining the appropriateness of intubation due to the lack of consensus regarding diagnosis, grading or prognosis of inhalation injury [12]. As such, decisions to intubate are rarely made without direct consultation with burns centres in Australasia from experienced prehospital and rural medicine practitioners who have been appropriately trained in airway assessment and management [24]. However, existing methods of predicting inhalation injury even by experienced practitioners are limited, largely relying on ‘soft’ or subjective clinical signs [25,26,27].

The findings of this study are comparable to a recent UK study [11], whereby 95% of their patient cohort of 40 intubated burn patients met ABA/Denver criteria for intubation and 30% developed VAP. This study, carried out in Manchester, proposed that the proportion of patients (30%) extubated within 48 h could represent potentially ‘avoidable’ intubations. Therefore, despite application of intubation scoring tools concerns remain regarding ‘over’-intubation and associated complications. Measuring the appropriateness of intubation by number of days ventilated is a problematic outcome measure in burn patients who may be appropriately intubated for shorter time frames if their indication is injury that primarily affects upper rather than lower airway. Extrapolation of conclusions drawn from results reported in European and US burn centres, aimed at minimising overzealous intubation, may not be applicable to Australasian burn care given the risks associated with loss of airway control during patient transfer to specialised burns centres over longer geographic distances. For instance, the RAH is a Level 1 trauma centre and a major burn referral centre with an extensive catchment area of 2.4 million square kilometers encompassing the entire state of South Australia and Northern Territories extending into neighboring regions in the states of New South Wales, Victoria, Western Australia. Approximately 40% of burn referrals to the RAH originate from rural and remote settings rather than from its immediate metropolitan catchment. This explains why a significant proportion of the present study cohort (*n* = 21, 38%) were intubated due to safety concerns relating to patient transport on retrieval to the RAH burns centre, often over long geographic distances. In contrast, burn centres in the UK such as Manchester [11] has a geographically smaller catchment area with shorter retrieval times and a higher density of trauma centres compared with many Australian/New Zealand burn centres.

Conventionally, the diagnosis of inhalation injury relies primarily on a history of smoke exposure in confined spaces, and/or physical findings such as those listed in the ABA and Denver Criteria (Table 1) [9,10,23]. Nasoendoscopy, laryngoscopy and bronchoscopy are valuable adjuncts for selection of burn-injured patients who can be safely managed without intubation. However, these are not universally available modalities particularly in pre-hospital settings. Despite the high proportion of patients meeting multiple ABA/Denver criteria for intubation in our results, 16 patients had normal findings with no evidence of lower-airway inhalation injury on bronchoscopy. Upper-airway inhalational injury may not be associated with any abnormal lower-airway findings on bronchoscopy and diagnosis of systemic inhalation injury can be challenging to diagnose but may also require ventilatory support. Thus, bronchoscopy (where available) may serve as a tool to confirm and grade severity of some inhalational injuries. However, it is not possible to diagnose or definitively outrule inhalation injury at a single time-point using this modality and serial examination along with interpretation of clinical findings is required. RSBI [15,16,23] does not appear to reliably predict successful extubation in our small study cohort given 13% of patients failed extubation, all of whom had RSBI scores well below the threshold of <105 for prediction of extubation success. This scoring system was generated for a general ICU patient cohort and is not burns specific; therefore, it may be of limited use for patients intubated following burn injury. Further study into the accuracy of scoring systems to predict successful extubation following burn injury could be valuable in reducing the number of days mechanically ventilated and overall ICU length of stay.

The mean RSBI score was 31 (range 12–61) in the remaining 49 patients who underwent successful endotracheal tube extubation at a mean of 3.3 days (Rage 3 h to 21 days). RSBI was originally reported by Yang et al. to have a sensitivity of 97% and a specificity of 64%. As such, RSBI performs better at predicting extubation success than it does at predicting failure of extubation. However, a 2001 meta-analysis by Meade et al. 26, reported poorer pooled sensitivity and of 84% and 44%, respectively. Subsets of patients who are more likely to have a false negative or inflated RSBI include (29): female gender, smaller endotracheal tube size and active closed system suctioning which causes a respiratory rate increase and tidal volume decrease, artificially inflating the calculated RSBI.

Thirteen patients (24%) developed VAP in this study cohort, the vast majority of whom were intubated for >48 h, which is well reported to be associated with burn sepsis, morbidity and mortality [28,29,30]. The United States Army Institute of Surgical Research reported that mortality appeared to increase by 20% in the presence of inhalation injury and by as much as 60% when inhalation injury and pneumonia coexisted. The effect of inhalation injury and pneumonia on mortality were found to be both independent and additive.

The RAH is a protocolised burns unit with a policy of immediate burn debridement and early burn wound closure using autologous skin or a dermal substitute at approximately 48 h post-burn depending on TBSA, burn wound depth and general physiological fitness for acute donor site harvest. The pathophysiology of inhalation injury has been described in detail in a previous paper by a co-author of this publication [31]. Early burn eschar excision allows for exploitation of an anaesthetic ‘window of opportunity’ before airway oedema and chemical pneumonitis become established. Thus, early burn excision policies are believed to confer major advantages in terms of respiratory physiology and facilitate early extubation (<48 h) compared with delayed debridement policies that may be associated with longer mechanical ventilation duration and progression to tracheostomy [30]. Multicentre studies using burn registry data are warranted to investigate the relationship between indications for endotracheal intubation and timing of burn debridement on the duration of mechanical ventilation, which may provide further evidence in support of early burn debridement protocols.

Limitations of this study include its retrospective nature, small sample size and failure to identify patients who were not intubated despite meeting ABA/Denver criteria—this population may be a cohort in whom clinicians use their judgment to avoid unnecessary intubations. The small sample size limits our findings in terms of determining the relationship, if any, between bronchoscopy findings and clinical criteria. Clinical scoring is a more practical tool than airway endoscopy to guide appropriate early intubation. The ABA criteria were initially introduced to address concerns regarding ‘unnecessary’ intubations and have been incorporated in the International Society for Burn Injury (ISBI) practice guidelines [10] with a reported sensitivity of 77% and specificity of 46% for detection of inhalational injury. Badulak et al. in Denver [23] modified the ABA criteria with the inclusion of two additional criteria, increasing its sensitivity to 95% but reducing specificity to 24% for prediction of long-term ventilation following intubation.

## 5. Conclusions

Most adult patients (95%) with burns admitted to the RAH are intubated per the ABA and Denver clinical criteria. Although current criteria may be oversensitive for diagnosing inhalation injury, this should be kept in context in terms of the burns system to which they are being applied, particularly given the potential hazards of failure to obtain a secure airway in patients with unrecognised evolving airway oedema or where transport times to specialised assessment are prolonged. Early nasoendoscopy, laryngoscopy and bronchoscopy are useful tools in predicting those who can be safely extubated <48 h. Serial airway examination using these modalities is required to inform decision making regarding the need for intubation due to the rapidly changing airway anatomy and dynamics associated with suspected inhalation injury, particularly for patients requiring major fluid resuscitation. Further research is required to overcome challenges that exist in defining inhalational injury and evaluating the appropriateness of endotracheal intubation in burn-injured patients.

## Figures and Tables

**Table 1:** American Burns Association and Denver indications for intubation of patients with suspected inhalation injury [9,10].

ABA Criteria [9] (2018)	Denver Criteria [10] (2018)
Signs of airway obstruction—hoarseness, stridor, accessory muscle use, and sternal retraction	Any of the ABA criteria listed with two additional indications below
Extent of burn > 40% TBSA	Singed facial or nasal hair
Extensive facial burns—deep dermal or full thickness depth	Suspected smoke inhalation
Dysphagia	
Intra-oral burns	
Signs of respiratory compromise—respiratory fatigue, hypoxia, and poor ventilation	
Reduced level of consciousness (LOC) with loss of protective reflexes	
Anticipated transfer of patient with major burn to burn centre without qualified medical professional to intubate en route	
Significant risk of oedema which may compromise airway	

**Table 2:** Intubated burn patient demographics and characteristics.

Demographic/Characteristic	Value	
Total Patients, *n*	55	
Mean Patient Age, years	48.2 (16–84)	
Male:Female	3:1 (*n* = 44 Male, *n* = 14 Female)	
Mortality, *n (% of total cohort)*	5 (9%)	
Location of Intubation, *n*	Pre-RAH—32	Pre-Hospital—21
		Other Hospital—11
	RAH—23	
Median Total Length of Acute Hospital stay	40 days (range 1–258)	
Median Length of ICU stay	4 days (range 1–50)	
Mean Ventilated days	5 days (range 0–39)	
Median TBSA %	20% (range 1–80)	
Mean APACHE II Score	15 (range 2–30)	
Mean APACHE III-j Score	53 (range 13–122)	

ICU = intensive care unit. TBSA = Total Body Surface Area of burn. APACHE = Acute Physiology and Chronic Health Evaluation Score, validated for mortality prediction in intensive care and burn patient populations [19,20,21,22].

**Table 3:** ABA and Denver indications for intubation [9,10].

	All Patients	Extubated within 48 h	Extubated from 48 h to 7 Days	Extubation > 7 Days
Single indication	7	4	2	1
Multiple indications	48	27	10	11
Total	55	31	12	12

**Table 4:** Bronchoscopy findings by the Abbreviated Injury Score (AIS) grading scale [13].

AIS Grade	0	1	2	3	4	Total
Grade Description	No injury	Mild injury	Moderate injury	Severe injury	Massive injury	
Patient numbers	16	4	3	1	0	24

AIS grading scale outlined in Supplementary material.

**Table 5:** Causes and demographics of patients with failed attempts to extubate.

Patient	TBSA and AIS Grade	Cause of Extubation Failure	VAP	Day Post-Burn at Failed Extubation	Day Post-Burn at Successful Extubation	RSBI
1.	60% TBSA with Grade 1 injury	Immediate oxygen desaturation	N	Day 3	Day 7	44
2.	7% TBSA with Grade 2 injury	Respiratory distress and tachypnea	N	Day 3	Day 12	28
3.	60% TBSA with Grade 1 injury	Respiratory distress and tachypnea	N	Day 5	Day 6	40
4.	7% TBSA with Grade 2 injury	Agitation and excessive respiratory secretions	Y	Day 4 and Day 9	Tracheostomy decannulated Day 14	63
5.	3% TBSA with Grade 0 injury and reduced GCS (Diffuse axonal injury)	Oxygen desaturation and excessive respiratory secretions	Y	Day 5	Tracheostomy decannulated Day 38	37
6.	20% TBSA with Grade 1 injury	Facial swelling and respiratory secretions	Y	Day 1	Day 4	31
7.	36% TBSA with Grade 0 injury and reduced GCS (Alcoholic Encephalopathy)	Poor spontaneous respiratory effort on attempted weaning	N	Day 1	Day 4	37

VAP = ventilator-associated pneumonia. RSBI [15] = rapid shallow breathing index. The ratio was determined by the breaths per minute divided by the tidal volume. An RSBI < 105 has been reported to predict successful weaning to extubation but is not the sole determinant of suitability for extubation. AIS [13] = Abbreviated Injury Score.

## Supplementary Materials

The following supporting information can be downloaded at: https://www.mdpi.com/article/10.3390/ebj4020014/s1. Table S1: Abbreviated injury score 13 (AIS) grading scale of inhalation injury on bronchoscopy.
