# Supplementary material for: Review of Indications for Endotracheal Intubation in Burn Patients with Suspected Inhalational Injury
Source: Eur Burn J. 2023 Mar 29;4(2):163–72. doi: 10.3390/ebj4020014 (PMC11571857; doi:10.3390/ebj4020014)
Supplement: Supplementary file 1 [file ebj-04-00014-s001.zip › ebj-2242587-supplementary.pdf]

european burn journal

Supplementary Material

Table S1. Abbreviated injury score [13] (AIS) grading scale of inhalation injury on bronchoscopy.

Grade	Class	Description
0	No injury	Absence of carbonaceous deposits, erythema, oedema, bronchorrhea, or obstruction
1	Mild injury	Minor or patchy areas of erythema, carbonaceous deposits, bronchorrhea, or bronchial obstruction
2	Moderate injury	Moderate degree of erythema, carbonaceous deposits, bronchorrhea or bronchial obstruction
3	Severe injury	Severe inflammation with friability, copious carbonaceous deposits, bronchorrhea, or obstruction
4	Massive injury	Evidence of mucosal sloughing, necrosis, endoluminal obstruction
